# Adhesion of Mesenchymal Stem Cells to Glycated Collagen—Comparative Analysis of Dynamic and Static Conditions

**DOI:** 10.3390/polym17060821

**Published:** 2025-03-20

**Authors:** Regina Komsa-Penkova, Anika Alexandrova-Watanabe, Svetla Todinova, Violina Ivanova, Svetoslava Stoycheva, Petar Temnishki, Borislav Dimitrov, Dobromir Dimitrov, Pencho Tonchev, Galya Georgieva, Aleksandar Kukov, Izabela Ivanova, Tihomir Tiankov, Emilia Abadjieva, Velichka Strijkova, George Altankov

**Affiliations:** 1 Department of Biochemistry, Medical University Pleven, 5800 Pleven, Bulgaria; violina.ivanova@mu-pleven.bg (V.I.); petar.temnishki@mu-pleven.bg (P.T.); borislav.dimitrov@mu-pleven.bg (B.D.);; 2 Leonardo da Vinci Center of Competence in Personalized Medicine, 3D and Telemedicine, Robotic and Minimally Invasive Surgery, 1, “St. Kl. Ochridski” Str., 5800 Pleven, Bulgaria; 3 Institute of Mechanics, Bulgarian Academy of Sciences, 1113 Sofia, Bulgaria; anikaalexandrova@abv.bg (A.A.-W.);; 4 Center of Competence at Mechatronics and Clean Technologies—MIRACle, “Acad. G. Bontchev” Str. 4, 1113 Sofia, Bulgaria; 5 Institute of Biophysics and Biomedical Engineering, Bulgarian Academy of Sciences, 1113 Sofia, Bulgaria; todinova@abv.bg; 6 Department of Oncology, Medical University Pleven, 5800 Pleven, Bulgaria; 7 Department of Surgery, Medical University Pleven, 5800 Pleven, Bulgaria; 8 Laboratory of Clinical Immunology, University Hospital “Lozenetz”, 1407 Sofia, Bulgaria; 9 Institute of Optical Materials and Technologies, Bulgarian Academy of Sciences, 1113 Sofia, Bulgaria; vily_strij@abv.bg; 10 Research Institute, Medical University Pleven, 5800 Pleven, Bulgaria

**Keywords:** mesenchymal stem cell (MSC), glycated collagen, dynamic and static adhesion, cell–matrix interactions, AFM

## Abstract

Understanding mesenchymal stem cell (MSC) behavior on glycated collagen is crucial for advancing regenerative medicine and understanding pathological mechanisms in diseases such as diabetes, cancer, and aging. While previous research has demonstrated reduced MSC interaction with glycated collagen under static conditions due to disrupted integrin signaling, these studies did not accurately replicate the dynamic mechanical environment that MSCs encounter in vivo. Here we present a comprehensive investigation comparing adipose-derived MSC (ADMSC) behavior under both dynamic flow conditions and static adhesion, revealing unexpected temporal dynamics and challenging existing paradigms of cell–matrix interactions. Using a sophisticated microfluidic BioFlux system combined with traditional static adhesion assays, we examined ADMSC interactions with native collagen for 1-day glycated (GL1), and 5-day glycated (GL5) samples. Under flow conditions, MSCs demonstrated remarkably rapid attachment—within 3–5 min—contrasting sharply with the classical 2 h static incubation protocol. This rapid adhesion was particularly enhanced on 5-day glycated collagen, though subsequent testing revealed significantly weaker adhesion strength under shear stress compared to native collagen. Static conditions also showed a distinct pattern: increased ADMSC adhesion to glycated samples within the first 30 min, followed by a progressive decrease in adhesion and compromised cell spreading over longer periods. Atomic force microscopy (AFM) analysis revealed significant changes in collagen surface properties upon glycation. These included a substantial reduction in the negative surface charge (from ~800 to 600 mV), altered surface roughness patterns (Rrms varying from 3.0 ± 0.4 nm in native collagen to 7.70 ± 0.6 nm in GL5), and decreased elasticity (Young’s modulus dropping from 34.8 ± 5.4 MPa to 2.07 ± 0.3 MPa in GL5). These physical alterations appear to facilitate rapid initial cell attachment while potentially compromising long-term stable adhesion through traditional integrin-mediated mechanisms. This study provides novel insights into the complex dynamics of MSC adhesion to glycated collagen, revealing previously unknown temporal patterns and challenging existing models of cell–matrix interactions. The findings suggest a need for revised approaches in tissue engineering and regenerative medicine, particularly in conditions where glycated collagen is prevalent.

## 1. Introduction

The adhesion of stem cells to collagen is a significant area of focus in regenerative medicine and tissue engineering research [1]. Understanding the interactions between stem cells and collagen under various conditions, both static and dynamic, is critical for enhancing the outcomes of regenerative therapies and optimizing tissue engineering approaches. Mesenchymal stem cells (MSCs) are undifferentiated cells with the capability to differentiate into different cell types, such as osteoblasts, chondrocytes, adipocytes, and occasionally fibroblasts or myofibroblasts [2]. While they remain undifferentiated in their niches, MSCs migrate to injury sites to assist in tissue repair through differentiation as needed [3,4]. Additionally, they contribute to collagen synthesis and remodeling during mechanical stretching [5]. The interaction between MSCs and the extracellular matrix (ECM), especially its primary protein collagen, is a complex subject extensively studied. Collagen fibrils serve as scaffolds, providing support to mesenchymal stem cells and other cells within tissues, which is crucial for tissue repair [6].

Collagen undergoes natural glycosylation during post-translational processing due to modifications by glucosyl and galactosyl transferases [7]. Recent research highlights that enzymatic glycosylation plays a pivotal role in regulating collagen secretion, aligning collagen fibrils, and promoting protein oligomerization [8]. However, throughout its lifespan, collagen can acquire covalently bound sugars through a spontaneous non-enzymatic process, which is generally very slow [9]. This phenomenon is more pronounced in diabetic tissues due to high sugar levels. Over time, early glycation products, known as Amadori products, can further react to form advanced glycation end products (AGEs), which can cross-link collagen molecules [10]. This process contributes to tissue stiffening alongside non-enzymatic oxidation and increased cross-linking caused by overexpressed lysyl oxidase or Rho-associated protein kinase, as observed in cancer [11]. AGEs are considered a major factor in ECM hardening associated with aging, although other ECM proteins also play a role [10]. Cross-linking can bind collagen molecules together, restrict their alignment, and ultimately impact fibril assembly, leading to pathological conditions accompanying various diseases [11,12]. In these scenarios, stem cells might bind to glycated collagen through RAGE (receptor for advanced glycation end products), which specifically recognizes AGEs [13,14]. Although this mechanism is not well understood, it may play a role in environments where integrin-mediated binding is impaired [13,15,16]. However, there are limited data on the effects of glycation at its earlier stages before the formation of Amadori products, particularly regarding their interactions with stem cells in both dynamic and static conditions.

Recent findings indicate that MSCs show different adhesion behaviors to native and glycated collagen substrates under static conditions [17]. However, this does not accurately represent the dynamic environment that mesenchymal stem cells encounter in vivo. It is widely recognized that stem cells adhere to collagen mainly through integrin-mediated interactions. Integrins, key receptors for cell adhesion to collagen and other ECM components, are transmembrane proteins that bind to specific sequences in collagen molecules, such as the GFOGER (glycine–phenylalanine–hydroxyproline–glycine–glutamate–arginine) motif found in fibrillar collagens [18]. Upon binding, integrins cluster on the cell surface and recruit intracellular adaptor proteins, forming focal adhesions that link the extracellular matrix (ECM) to the cytoskeleton, a phenomenon known as integrin-dependent adhesion, based mostly on in vitro studies [18,19,20]. While integrins are crucial for cell adhesion and signaling during stem cell–collagen interactions, there are limited data on the kinetics of such adhesion under early glycation conditions. This is especially true when comparing dynamic conditions (adhesion under flow) to static conditions (adhesion driven by sedimentation). Research in this area is particularly important, as it has significant implications for regenerative medicine and for understanding pathological mechanisms associated with diseases like diabetes and aging [1,6].

## 2. Material and Methods

### 2.1. Collagen Preparation

**Figure 1:**
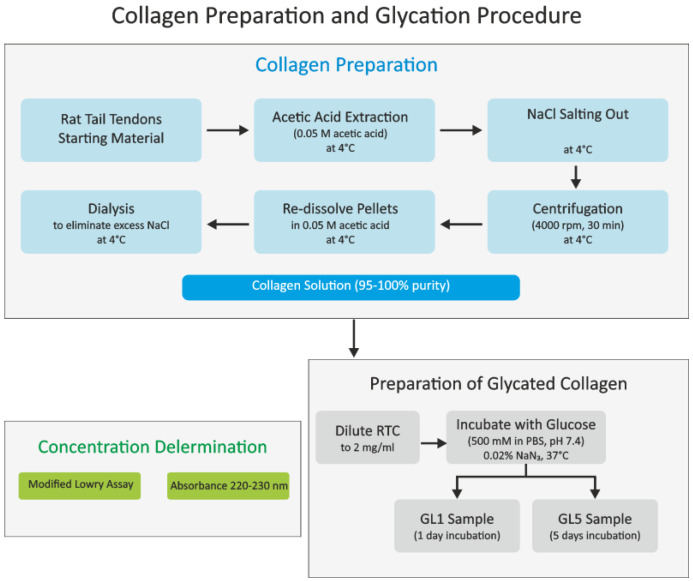
Collagen processing. Collagen was extracted from rat tendons by 0.5 M acetic acid and salted out with NaCl. The pellets were centrifugated, redissolved, and dialyzed versus 0.05 M acetic acid. Collagen samples (2 mg/mL) were glycated by incubating with a 500 mM glucose solution in PBS pH 7.4 for 1 (GL1) or 5 (GL5) days at 37 °C. To characterize the extent of glycation through free amino group quantification, collagen samples were redissolved in sodium bicarbonate at pH 8.5 and TNBS was added. The samples were visualized by adding SDS and 1 N HCl, and the absorbance was measured at 335 nm.

Collagen type I was extracted from rat tail tendons using acetic acid extraction followed by salting out with NaCl, as detailed in our prior studies (Figure 1) [17]. The resulting pellets were collected through centrifugation at 4000 rpm (RCF 2650× *g*) for 30 min at 4 °C, then redissolved in 0.05 M acetic acid and dialyzed to eliminate excess NaCl. This process yielded a collagen solution with collagen content nearing 95–100% of the total dry mass. All steps were conducted at 4 °C. The concentration of collagen in the solutions was determined using a modified Lowry assay [21] and by measuring optical absorbance at 220–230 nm [22].

### 2.2. Preparation of Glycated Collagen

Rat tail tendon collagen (RTC), obtained as described above and diluted to a concentration of 2 mg/mL, was glycated by incubating it with a 500 mM glucose solution (Merck) in PBS at pH 7.4 with 0.02% NaN_3_ for either 1 or 5 days at 37 °C, following a prior protocol (Figure 1) [17]. The samples were designated GL1 and GL5 after being dialyzed versus 0.05 M acetic acid and stored at 4 °C until further use. The quantification of free amino groups (characterizing the extent of glycation) in collagen samples was performed using the 2,4,6-trinitrobenzene sulfonic acid (TNBS) method, based on Fields’ original procedure [23]. Briefly, collagen samples (0.5 mL) were dissolved in 0.1 M sodium bicarbonate at pH 8.5 at a concentration of 100 μg/mL. A freshly prepared solution of 0.01% (*w*/*v*) TNBS (0.25 mL) (Thermo Fisher Scientific, Waltham, MA, USA) was added, and the samples were incubated at 37 °C for 2 h. The reaction was visualized by adding 0.25 mL of 10% SDS and 0.125 mL of 1 N HCl to each sample, and absorbance was measured at 335 nm. Quantitative determination of amine content in the samples was achieved compared to a standard curve created using glycine (2–20 μg/mL).

### 2.3. Cells

Human adipose tissue-derived mesenchymal stem cells (ADMSCs) at passage 2 were obtained from the Tissue Bank BulGen, with written consent from volunteers prior to liposuction. The cells were cultured in DMEM/F12 medium supplemented with 1% GlutaMAX™, 1% antibiotic–antimycotic solution, and 10% fetal bovine serum (FBS), all sourced from Sigma Aldrich. The medium was changed every two days until the cells reached approximately 90% confluence, after which they were used for experiments up to the seventh passage. The viability of the ADMSCs was checked using the trypan blue exclusion test upon cell harvesting, with a viability rate of over 85% considered acceptable for all experiments.

### 2.4. Cell Adhesion Studies

#### 2.4.1. Static Adhesion Assay

For the static adhesion studies, collagen (native and glycated samples (100 μg/mL) dissolved in 0.05 M acetic acid) were used to coat regular glass coverslips (12 × 12 mm, ISOLAB Laborgeräte GmbH, Eschau, Germany) via incubation for 60 min at 37 °C in 6-well TC plates (Sensoplate, Greiner Bioone, Meckenheim, Germany). After washing 3 times with PBS, cells were seeded at a density of 5 × 10^4^ cells/well in a final volume of 3 mL serum-free medium and incubated for various periods (15, 30, 60, and 120 min). Different plates were used for various periods of adhesion. Adherent cells were visualized using fluorescein diacetate (final concentration of 5 μg/mL in PBS (stock solution of 5 mg/mL in acetone) after gentle washing of unattached cells with PBS. Each sample was duplicated so that 10 representative images could be taken per variant using an inverted fluorescent microscope (Leica DM 2900) at 20× magnification before adhering cells being counted manually. The whole experiment was conducted 2 times with similar results. The data presented are from the second experiment.

For more detailed morphological examination, samples were cultured under identical conditions on native and glycated collagen-coated glass coverslips for either 30 min or 2 h in a serum-free medium. The overall morphology of ADMSCs was studied at 63× magnification using an inverted microscope (Leica DM 2900) after being processed for immunofluorescence staining and morphometric analysis. More specifically, following incubation, samples were fixed with 4% paraformaldehyde and permeabilized with 0.5% Triton X-100 before staining. Green fluorescent Alexa Fluor™ 488 phalloidin (Invitrogen, Thermo Fisher Scientific Inc., Branchburg, NJ, USA) was used to visualize the actin cytoskeleton. Cell nuclei were stained with Hoechst 33342 (dilution 1:2000) (Sigma-Aldrich/Merck KGaA, Darmstadt, Germany). while focal adhesions were detected with anti-vinculin mouse monoclonal antibody (clone: hVIN-1, Thermo Fisher Scientific, Waltham, MA, USA) IgG, diluted 1:100, followed by fluorescent Alexa Fluor 555-conjugated goat anti-mouse IgG secondary antibody (Sigma-Aldrich), also diluted 1:100.

#### 2.4.2. Dynamic Adhesion Assay

##### Microfluidic System and Experiments

**Figure 2:**
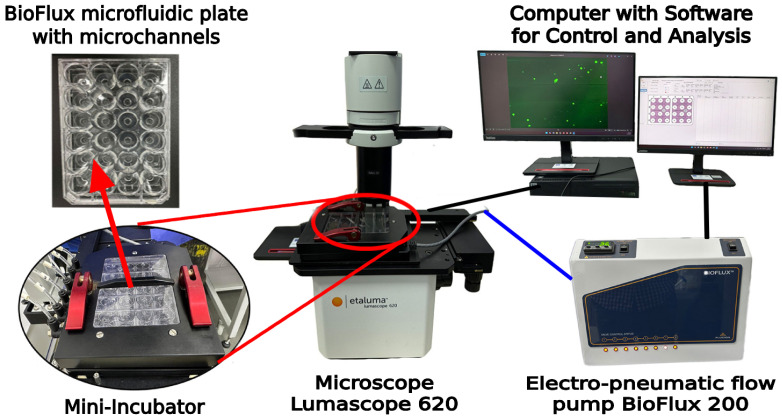
Schematic representation of experimental setup of BioFlux microfluidic system, comprising an electro-pneumatic flow control pump BioFlux 200 (Fluxion Biosciences, Oakland, CA, USA), an inverted fluorescence microscope Lumascope 620 (Etaluma, San Diego, CA, USA), a BioFlux microfluidic plates (Fluxion Biosciences, Oakland, CA, USA) with microchannels, and a computer configuration with specialized control and analysis software.

To study the adhesion and detachment of stem cells in flow, we used the BioFlux system (Fluxion Biosciences, Oakland, CA, USA), a high-quality platform for in-flow rheological analyses driven by air pressure. The setup includes a BioFlux 200 (Fluxion Biosciences, Oakland, CA, USA) electro-pneumatic flow control pump, a BioFlux microfluidic plate (Fluxion Biosciences, Oakland, CA, USA) placed in a temperature control system fitted to an inverted fluorescence microscope (Lumascope 620Etaluma, San Diego, CA, USA), and a computer configuration with control and analysis software (Figure 2). For the experiments, we used corresponding BioFlux 24-well plates with 0–20 dyn/cm^2^, consisting of 8 microfluidic channels, each featuring two input wells and one output well, with cross-sectional dimensions of 350 μm in width and 75 μm in height.

##### Coating the Microfluidic Channels with Collagens

Each of the three outlet wells was filled with 200 μL of prepared solutions of native collagen, glycated collagen for 1 day (GL1), and glycated collagen for 5 days (GL5). The concentration was 100 µg/mL in 0.05 M acetic acid in water. These collagen solutions were perfused through the microfluidic channels at a shear stress of 5 dyn/cm^2^ for 3 min, after which the channel flow was abruptly stopped. The microfluidic channels were incubated at 37 °C for 60 min. Excess collagen solution was aspirated from the inlet and outlet wells and replaced with distilled water and a second PBS solution. The channels were washed at 1 dyn/cm^2^ shear stress for 3 min.

##### Design of the Experiment for Adhesion of Stem Cells to Collagen-Coated Microchannels

A 0.5 mL stem cell solution in PBS, with a concentration of 5 × 10^6^ cells/mL (as determined by an EVE™ Automated Cell Counter, NanoEnTek), was mixed with 0.5 mL of Histopaque-1077. To this 1 mL solution, 10 µL of calcein AM was added at a concentration of 1 mM in DMSO (dimethyl sulfoxide). From the resulting solution, 330 µL was added to each of the three wells containing collagen-coated microchannels. The stem cell solution was perfused through the microfluidic channels at a shear stress of 2 dyn/cm^2^ for 1 min; then, the flow was reduced to 0.2 dyn/cm^2^ for 30 min at 37 °C. At least four images of adherent ADMSCs were captured every 5 min along the entire visible length of the channel at randomly selected locations for the three types of collagen samples.

##### Design of the Experiment for Detachment of Stem Cells from Collagen-Coated Microchannels

The flow was abruptly halted after 30 min of stem cell adhesion to the collagen-coated microchannels. The excess stem cell solution was aspirated from the inlet and outlet wells, followed by adding PBS solution to the outlet well. The microchannels were then washed twice at a shear stress of 0.4 dyn/cm^2^ for 3 min each. Subsequently, 1 mL of PBS solution was added to each outlet well, and the shear stress was incrementally increased every 3 min to 1, 5, 10, 15, and 20 dyn/cm^2^. At each shear stress level, at least four images of detaching stem cells were captured along the entire visible length of the channel at randomly selected locations.

The experiments for adhesion and detachment of stem cells to the three types of collagen were repeated three times with similar results. The data, presented as means ± SD, are from the last experiment.

### 2.5. Atomic Force Microscopy Measurements

AFM imaging of native and glycated collagen was accomplished using atomic force microscopy (MFP-3D, Asylum Research, Oxford Instruments, Santa Barbara, CA, USA). The measurements were conducted in tapping mode for surface morphology and surface potential and in contact mode for the evaluation of Young’s modulus and adhesion forces at room temperature. Contact mode scanning was performed using conical silicon AFM tips (Nanosensors, type qp-Bio, Neuchatel, Switzerland), with a resonance frequency of 50 kHz, a nominal spring constant of 0.3 N/m, and a nominal tip radius of 8 nm.

In the AFM experiments, collagen solutions were first applied to glass coverslips and incubated for one hour at 37 °C. The collagen-coated coverslips were then carefully rinsed with distilled water to prevent crystallization of the buffer on the surface.

Roughness analysis was performed in a scanning area of 5.0 µm × 5.0 µm. The Rrms value was calculated as the mean square root of the height distribution as follows:

$$Rrms=\sqrt{1/N\sum_{i=1}^{N}z_{i}^{2}}$$

where *z_i_* is the height at a given pixel *I* and *N* is the total number of pixels in the image.

The force mapping was achieved on a grid of 16 × 16 points. The value of Young’s modulus was obtained by fitting the force–distance curves to the Hertz model with the embedded IgorPro 6.37 software, considering the Poisson’s ratio (υ) to be 0.5:

$$E_{a}=3F(1-\upsilon^{2})/4\sqrt{{r\delta }^{3}}$$

where *F* is the applied force on the sample, *δ* is the indentation depth, *r* is the tip radius, and *E* and *υ* are Young’s modulus and Poisson’s ratio, respectively.

The adhesion forces arising from the interaction between the AFM probe and the surface of the sample were recorded as adhesion maps simultaneously alongside indentation force maps during AFM experiments.

Scanning Kelvin probe microscopy (SKPM) was employed for measuring potential maps of samples. SKPM measurements were performed in non-contact AC mode using conductive (Ti/Ir coated) tips with the resonance frequency of 70 kHz and an elastic constant of 2 N/m.

### 2.6. Statistical Analysis

The entire experiment on static adhesion was conducted twice with similar results. Each sample was duplicated so that 10 representative images could be taken per variant before adhering cells were counted manually.

The experiments for dynamic adhesion and detachment of stem cells to the three types of collagen samples were also repeated three times. Data are presented as means ± SD (standard deviation). A non-parametric Wilcoxon test was used to compare data between independent groups with non-Gaussian distribution. Significant differences were considered at the level of *p* ≤ 0.05.

One-way analysis of variance (ANOVA) followed by Tukey HSD post hoc tests were performed on all other datasets. Error is shown in bar graphs as mean ± SD unless otherwise noted. Significance is indicated by asterisks, corresponding to *p* < 0.05.

## 3. Results

### 3.1. Adhesion Behavior of Stem Cells to Collagen in Flowing Conditions

The experimental design for these studies focused on assessing the adhesion and detachment of stem cells under flow, utilizing the air pressure-driven microfluidic system, BioFlux. This system is a state-of-the-art imaging platform tailored for conducting the adhesive behavior of living cells placed in specially designed microchannels. The microchannels were coated with various types of collagen, including native type I collagen sourced from rat tails (RTC Nat), as well as the samples of collagen—glycated for 1 day (GL1) and 5 days (GL5)—each coated via incubation for 1 h, followed by excessive washing with PBS, as described in the Methods section.

### 3.2. Dynamic Adhesion of Stem Cells

**Figure 3:**
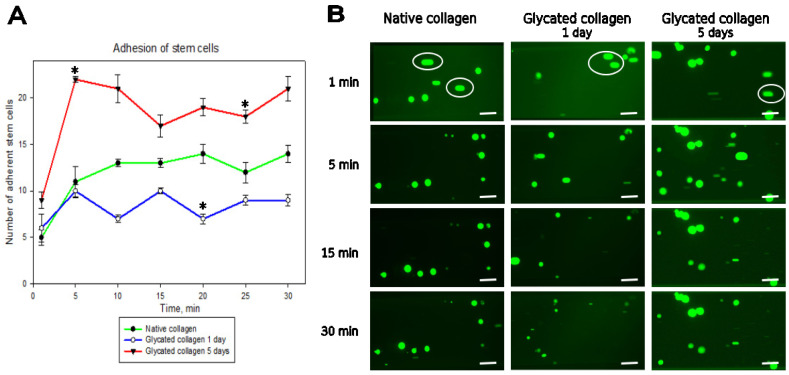
Dynamic adhesion of stem cells to glycated collagens at constant flow shear stress of 0.2 dyn/cm^2^. (**A**) Change in the average number of stem cells adhering to the microchannels coated with either native (green line) or glycated collagens for 5 days (GL5, red line) or 1 day (GL1—blue line), respectively. (**B**) Representative images of adhering cells viewed with calcein AM before counting at 1 min, 5 min, 15 min, and 30 min. The images exemplify approximately one-fifth of the visual field of the channels. The white ellipses show the non-adhering cells, which look “elongated” because they move with the flow during the image capture. They appeared mostly in the 1st minute and were not counted when determining the average number of adhered stem cells. * Statistically significant difference (*p* < 0.05) in the values of number of stem cells adhering to GL1 and GL5 compared with native collagen. Scale bar—50 µm.

Adhesion of ADMSCs under flow was assessed at a tangential flow of 0.2 dyn/cm^2^ for 30 min. As shown in Figure 3A, adhesion began quickly around the 5 min mark, reaching its peak for the samples glycated for 5 days (red line), followed by adhesion to native collagen (green line). Adhesion was lower for 1-day glycated samples (blue line), and this pattern persisted throughout the 30 min duration of the experiment, with little additional cell attachment observed. Representative images of adhering cells to the microchannels are presented in Figure 3B.

### 3.3. Detachment Kinetic of ADMSCs Under Flow

To learn more about the strength of stem cells’ adhesion to the different collagen substrata, the adhering cells (at a flow of 0.2 dyn/cm^2^ for 30 min) were subjected to stepwise increasing shear stress from 0–2 to 20 dyn/cm^2^ [24]. It has to be noted that in the bloodstream, stem cells are subjected to varying levels of shear stress, typically ranging from 0.5 to 20 dyn/cm^2^. For example, shear stress of around 20 dyn/cm^2^ is generally observed in arteries due to the high flow rates and pressure, though physiological shear stress can range from 0.5 to 120 dyn/cm^2^ and is highest in small arterioles and capillaries [25].

**Figure 4:**
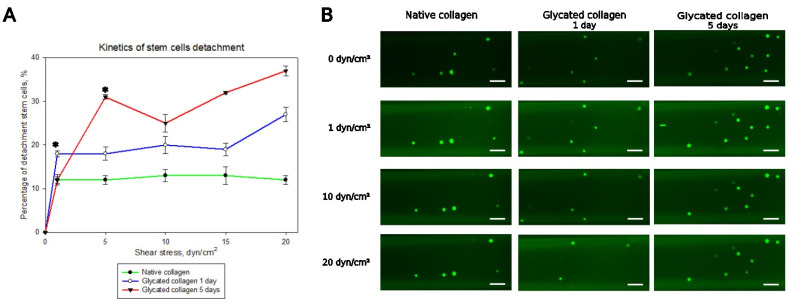
Stem cell detachment. (**A**) Variation in the percentage of MSC detachment from channels coated with native collagen, glycated collagen GL1 (1 day), and GL5 (5 days) at shear stress of 1 dyn/cm^2^, 5 dyn/cm^2^, 10 dyn/cm^2^, 15 dyn/cm^2^, and 20 dyn/cm^2^. (**B**) Representative images of one-fifth of the visual field of the channels, showing some stem cells still adhering after the application of the abovementioned shear stresses. * Statistically significant difference (*p* < 0.05) in the values of stem cells adhering to GL1 and GL5 compared with native collagen. Scale bar—50 µm.

Our model experiments for detachment were performed at a range of physiological shear stress of 0 to 20 dyn/cm^2^ (namely: 0, 1, 5, 10, 15, and 20 dyn/cm^2^), which is characteristic of big arteries, each applied for 3 min. The representative detachment curves are shown in Figure 4. Figure 4A shows that the initial detachment of stem cells from native collagen (indicated by the green line) remains relatively stable after the initial wash at 1 dyn/cm^2^. Native collagen and GL5 presented 12% detachment of stem cells, but GL1 had ~18% detachment at 1 dyn/cm^2^. For the next shear stresses (5, 10, and 15 dyn/cm^2^), detachment was significantly higher for the glycated collagen samples. For the 1-day glycated samples (blue line), cell detachment increased by approximately 19%, while for the 5-day glycated samples (red line), an increase of 30% was observed. At the highest shear stress (20 dyn/cm^2^) in native collagen, stem cell detachment remained at 12%, in contrast to GL1, where detachment increased to 27%, and at GL5, it reached the largest increase of 37%. Representative images of cells adhering to the bottom of microchannels shown in Figure 4B support this conclusion.

### 3.4. Static Adhesion Assay of ADMSC

This study focused on the initial stages of ADMSC attachment (within the first 2 h), as it was anticipated that at later stages, cells would generate a variety of matrix proteins, potentially complicating adhesion exclusively to collagen. For this reason, the adhesion was conducted in a serum-free medium to avoid competition from other proteins. For the static adhesion assay, standard 24-well tissue culture plates were precoated with either native (Nat) or glycated collagens GL1 or GL5 under the same conditions as previously described. ADMSC adhesion was evaluated by counting the adherent cell numbers at specified time points (15, 30, 60, and 120 min). Live cells were stained with fluorescein diacetate (FDA) shortly before being imaged using an inverted fluorescent microscope. At least 10 representative images were captured from each well for statistical analysis.

**Figure 5:**
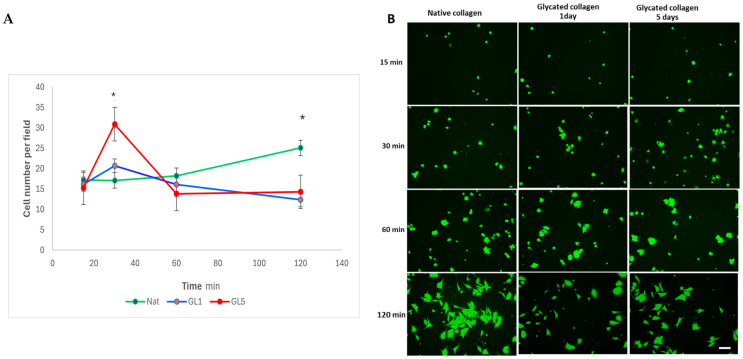
Kinetics of ADMSC adhesion on native (Nat), 1-day glycated (GL1) and 5-day glycated (GL5) collagen. (**A**) The kinetics of cell adhesion, represented as means ± SD of cells per slide (ordinate) in relation to incubation time in minutes. (**B**) Representative images of adhering living cells were viewed by FDA at different time points. * Statistically significant difference (*p* < 0.05) in stem cell numbers adhering compared to native collagen. Scale bar: 50 μm.

As highlighted in Figure 5A, after an almost equal initial cell attachment at the 15 min mark, ADMSC adhesion to native collagen samples moderately increases, reaching about twice the initial attachment level by the second hour. Additionally, noticeable cell spreading is observed, particularly at 120 min (Figure 5B). On the other hand, adhesion to glycated collagens GL1 and GL5 rose sharply at 30 min, more than doubling for the 5-day glycated samples and showing a less pronounced increase of approximately 20% for the 1-day glycated samples. However, this initial peak of adhesion to glycated collagen declines significantly with further incubation, falling below the adhesion level of native collagen by the first hour and continuing to decrease by the second hour. Additionally, cells exhibit less spreading on glycated collagen samples by the end of the incubation period of 120 min.

### 3.5. Overall Cell Morphology at Early and Late Stages of Adhesion

For more detailed morphological studies, glass coverslips were coated with either native or glycated collagens using the same protocol described above. Given the surprisingly high adhesion of ADMSCs to glycated collagen within the first 30 min of incubation, cells were cultured on these collagen substrates for 30 min and 120 min to allow them to spread and develop their adhesive machinery. After these time periods, the cells were fixed and stained for actin, vinculin, and nuclei.

**Figure 6:**
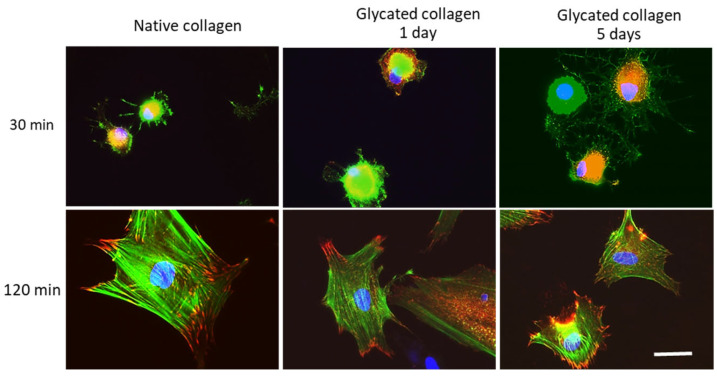
ADMSC spreading on different collagen substrata. Development of focal adhesions (red) and actin cytoskeleton (green), while cell nuclei are counterstained in blue. Bar 20 μm.

Figure 6 shows our overall morphological observations. After 2 h (bottom row), cells spread better on native collagen substrates (first left image), developing well-pronounced focal adhesion contacts, where actin stress fibers regularly insert.

A trend of diminished cell spreading was observed on glycated collagen samples depending on glycation extent. For 1-day glycated samples (middle section), ADMSCs appear smaller, but still develop focal adhesions and actin fibers almost normally. For 5-day glycated samples, the cells appear more shrunken, yet still express focal contacts and minimal stress fibers. Conversely, after a short 30 min incubation (upper panel), stem cells attach well and seem to spread more effectively on glycated collagen samples, although focal contacts and stress fibers were not observed in any substrates.

### 3.6. Alteration in Collagen Surface Morphology and Electrostatic Potential upon Glycation—AFM Study

**Figure 7:**
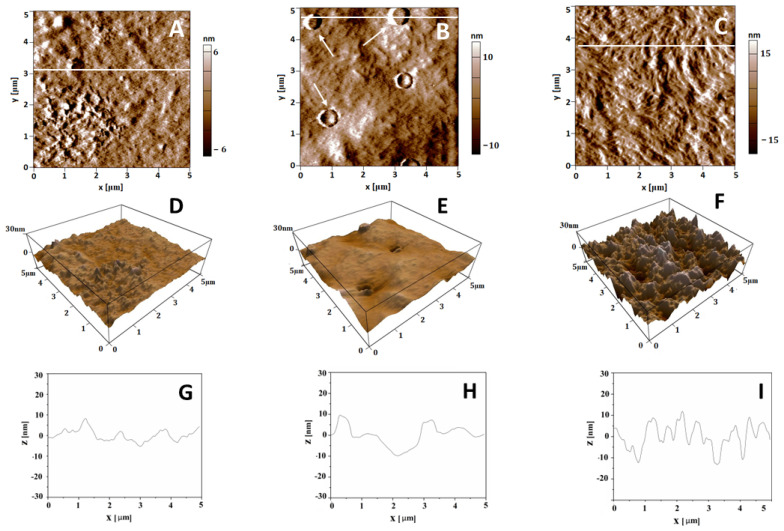
Representative 2D AFM images of native collagen (**A**), collagen glycated for 1 day (**B**), and collagen glycated for 5 days (**C**). 3D topographical images (**D**–**F**) corresponding to the images in panels (**A**–**C**). Cross-section plots (**G**–**I**) corresponding to the white lines in (**A**–**C**). The arrows in panel (**B**) indicate the circular aggregate formations. The scanned area is 5 × 5 μm^2^. All images were taken in tapping mode at room temperature.

In this study, we employed atomic force microscopy (AFM) to map a surface’s morphology, nanomechanical properties, and electrostatic potential of collagen upon glycation at nanometer resolution. As shown in Figure 7A,D, the surface of native collagen has a folded structure with a root mean square roughness (Rrms) of 3.0 ± 0.4 nm (calculated from topographic images with an area of 5 × 5 μm^2^), indicating a moderately rough surface at the nanoscale.

Glycation significantly alters the surface structure of collagen, with these changes closely related to the duration of the glycation process. AFM topographical images of collagen glycated for 1 day (GL1) show a smoother structure with an Rrms of 2.53 ± 0.3 nm (summarized in Table 1), along with the appearance of unusual clusters of circular filaments, which are absent in native collagen (Figure 7B). These clusters are approximately 300 nm in diameter and 5 nm in height and can result from changes in the molecular arrangement of collagen by glycation. When AFM data were analyzed from an area (2 × 2 μm^2^) where no such filament aggregates were detected, the Rrms value was even lower (1.75 ± 0.07 nm), indicating a smoother surface in these regions. The decrease in the membrane roughness is evident in the 3D image (Figure 7E), providing a more comprehensive view of the overall topography changes in the collagen surface after 1-day glycation. The cross-section plot (Figure 7H) defined by the white line in Figure 7B shows these aggregate formations.

The morphology of collagen glycated for 5 days (GL5) appeared rougher with an Rrms of 7.7 ± 0.6 nm, with more pronounced linear arrangements than the native collagen and the 1-day glycated collagen (Figure 7A,B). The overall thickness of the protein layer approximately doubled versus the native collagen, from 10 to about 20 nm.

On the other hand, as summarized in Table 1, the average surface potential (in mV) was determined to be 789 ± 93.7 mV for the native collagen, as calculated from the map of surface potential in the same scanned area). A substantial decrease in the average surface potential was observed for the GL1 collagen sample (559 ± 18.4 mV) (Table 1), indicating that glycation may alter the electrostatic properties of the collagen, potentially affecting its interactions with surrounding molecules. Additionally, it is noteworthy that the circular formations observed on the surface exhibited a higher surface potential than the areas between them (Figure 7B), suggesting that the initial glycation can lead to heterogeneous changes in the collagen surface. The surface potential of 5-day glycated collagen (659 ± 8.8 mV) was slightly higher than that of the 1-day glycated sample, but remained lower than that of the native collagen (Table 1).

### 3.7. Young’s Modulus and Adhesive Forces of Native and Glycated Collagens

In addition to collagen’s morphometric and electrical properties, AFM was used to analyze the forces acting between the AFM probe and the sample surface. By recording the deflection of the cantilever as it interacts with the sample, AFM quantifies forces such as adhesion and elasticity. The Young’s moduli (Ea) of the three modifications of collagen were determined from force–distance curves, providing insight into the mechanical properties of the samples. Ea reflects the stiffness or elasticity of the sample, with different values observed for native and glycated collagens at 1 day and 5 days. The Young’s modulus (Ea) of the native collagen was determined to be 34.8 ± 5.4 MPa (Table 1). A significant decrease in stiffness was observed in the glycated collagen samples depending on the duration of glycation. Surprisingly, Young’s modulus for GL1 was found to decrease to 6.90 ± 0.5 MPa, i.e., approximately five times lower than that of native collagen. The elastic modulus further decreased with increased glycation time. For GL5, Young’s modulus dropped to 2.07 ± 0.3 MPa, indicating a substantial reduction in stiffness with prolonged glycation under our experimental setup. This progressive decrease in Young’s modulus suggests that glycation in our conditions results in substantial softening (or reduced rigidity) of the collagen structure.

The measured adhesive forces for native collagen were 57.5 ± 0.6 nN (Table 1). Following the trend observed for Young’s modulus, glycation led to a decrease in adhesive forces, although less pronounced (Table 1). For 1-day glycated collagen, the adhesive forces significantly decreased to 31.14 ± 0.3 nN, considerably lower than native collagen. For 5-day glycated collagen, the adhesive forces further decreased to 17.06 ± 0.2 nN, indicating a continued reduction in adhesion strength as glycation progressed. This suggests that glycation affects the surface stickiness of collagen, likely due to changes in surface chemistry that alter collagen’s ability to interact with the AFM cantilever.

## 4. Discussion

In a previous study, we demonstrated that MSC interaction with glycated collagen is significantly reduced under static conditions, which we attributed to disrupted integrin signaling during the early stages (1–5 days) of collagen glycation [17]. However, the protocol used in that study did not accurately replicate the dynamic mechanical environment that MSCs encounter in vivo. Therefore, here we focused on examining the behavior of ADMSCs under dynamic conditions (e.g., under flow), while also studying static adhesion within the same time frame for comparison. Our findings revealed that under flow, MSCs attach to collagen remarkably quickly—within the first 3–5 min—contrasting sharply with the classical protocol of a 2 h incubation in static conditions we used previously. This rapid adhesion suggests that integrin-dependent attachment typically involves receptor clustering, focal adhesion formation, cytoskeleton development, and eventual cell polarization [17,19,26,27], and can occur much faster than previously thought. However, we also observed that dynamic adhesion to glycated collagen was significantly enhanced, particularly on samples glycated for 5 days. This pattern persisted for up to 30 min, with only minor deviations (Figure 3), though 1-day glycated samples did not exhibit the same trend. Kinetics of cell detachment (Figure 4) further revealed that ADMSCs actually adhered weakly to glycated collagen, as at a shear stress of 20 dyn/cm^2^, one-third of cells detached from 1-day glycated samples and three times more cells detached from 5-day glycated samples, whereas attachment to native collagen remained stable. This indicates that although glycated collagen promotes rapid adhesion, the strength of adhesion is compromised compared to native collagen.

The current findings from the comparative study of adhesion under static conditions also revealed a significant increase in ADMSC adhesion to 5-day glycated samples, approximately doubling within the first 30 min of incubation. A similar but less pronounced trend was observed for 1-day glycated samples, showing an increase of about 20% (Figure 5). However, prolonged incubation led to a decrease in adhesion to glycated collagen, falling below that of native collagen after the 1 h mark and continuing to decrease by the 2 h mark. As a result, cells spread less on glycated collagen than native collagen after 2 h, corroborating prior observations [17]. It is demonstrated here in the lower row of Figure 6.

Integrin-mediated interactions primarily involve αvβ3 and α5β1 integrins, which recognize the RGD motif in some collagens [19]. Specifically, the GFOGER sequence expressed in most collagens is recognized by α1β1, α2β1, α10β1, and α11β1 integrins [28,29,30]. These interactions are crucial for the later stages of adhesion, including forming focal adhesions and developing the actin cytoskeleton [26]. However, our findings suggest an alternative, presumably faster adhesion mechanism, during the early stages of adhesion before integrin clustering. The literature indicates that the receptor for advanced glycation end products (RAGE), which is constitutively expressed in stem cells [31], may facilitate rapid initial adhesion to glycated collagen [14,16,32,33,34]. RAGE, a cell adhesion molecule of the immunoglobulin superfamily, plays a key role in stem cell behavior in pathological environments like diabetes, cancer, and aging, often involving oxidative stress and inflammation [6,31,35]. Studies have shown that RAGE-expressing cells have increased adherence and spreading in the presence of ECM proteins and seem to adhere faster to collagen [13,14]. Therefore, RAGE is a primary candidate for enabling rapid initial adhesion of ADMSCs to glycated collagen. RAGE also shares significant similarity with other adhesive proteins, including MUC18 (melanoma cell adhesion molecule) and activated leukocyte cell adhesion molecule (ALCAM) [31,36]. This further suggests that AGE-induced alterations in stem cell adhesion behavior might be important in tissue engineering and regenerative medicine [1,37].

Despite the observed rapid initial adhesion to glycated collagen, integrin-dependent adhesion persisted, as ADMSCs formed focal contacts and showed polarized actin cytoskeletons at the 2 h incubation mark, though these were weaker compared to cells adhering to native collagen. Interestingly, within just a few minutes, cells adequately attached to both native and glycated collagens under dynamic conditions, despite the lack of focal adhesions and actin cytoskeleton development on either surface (shown in Figure 7). This suggests that adhesion of stem cells may occur faster than typically expected, particularly under flow conditions. Though we do not have direct proof of this, we believe that in such dynamic conditions, ADMSCs attach faster because of their rolling over the substratum, which exposes more adhesive receptors to the collagen molecules.

Another important factor involves the physical parameters of the cell adhesive environment and how glycation alters them. While the ligand–receptor theory for cell adhesion is widely accepted [26]—for example, coating a surface with adhesion molecules like collagen or fibronectin will necessarily increase adhesion—it is only one piece of the puzzle [38]. van der Waals forces, which are universally present between all molecules, must also contribute to cell adhesion, along with factors such as geometry, elasticity, and surface molecule distribution. Cell adhesion occurs when there is a balance between electrostatic repulsive forces and van der Waals forces of attraction. Therefore, cells first need to overcome the electrostatic barrier to reach the substratum. Our findings show that the negative surface charge diminishes substantially (by approximately 20–25%) upon collagen glycation, reducing from about 800 to 600 mV. This reduction facilitates faster adhesion, explaining the surprisingly quick initial attachment of ADMSCs to glycated collagen samples. This also correlated with a significant drop in the non-specific adhesive interaction with the AFM tip, decreasing approximately two- and threefold for 1-day and 5-day glycated samples, respectively (Table 1).

Apart from this, cell adhesion is also dependent on other key substratum parameters, such as roughness and elasticity [39]. Topographic AFM images showed that the surface of native collagen has a folded, fiber-like structure with a root mean square roughness (Rrms) of 3.0 ± 0.4 nm. However, glycation did not have a uniform impact: in 1-day glycated samples, it affected only specific areas with localized aggregates with a mean background Rrms of 2.53 ± 0.3 nm. One plausible explanation for the appearance of such localized aggregates at the early stages of glycation may be the uneven distribution of early glycation products. As glycation time increases, these changes started to affect the entire collagen structure, leading to an increased thickness of the adsorbed protein layer and considerably rougher morphology, with an Rrms of 7.70 ± 0.6 nm for 5-day glycated samples. Though we did not measure pre-AGEs and AGEs directly (as there is a lack of consistency because of the wide variety of compounds [10]), this finding suggests ongoing molecular changes. The increased roughness likely stems from complex molecular rearrangements, such as aggregation, which could alter cellular interactions in the later phases of adhesion. Generally, increased roughness supports cell attachment [36], which could explain the sharp increase in ADMSC adhesion to glycated collagen (around 30 min) before integrin clustering. As incubation progresses, we believe that integrin-dependent adhesion begins to dominate and stabilize adhesion through the formation of focal adhesion clusters and actin cytoskeleton development. However, this stabilization is not complete, as cells exhibit a shrunken morphology on glycated samples. This incomplete stabilization is likely due to the interplay of two processes—disrupted integrin-dependent adhesion and non-specific substrate-dependent attachment—which supports ADMSC anchorage. In terms of elasticity, Young’s modulus significantly decreased with the extent of glycation, surprisingly sharply dropping from 34.8 ± 5.4 MPa to 2.07 ± 0.3 MPa. This suggests a substantial reduction in stiffness (over tenfold), which may also account for diminished non-specific adhesion at later stages of incubation.

Although it is well established that the formation of AGEs in collagen typically leads to tissue stiffening through cross-linking [1,7,10], which generally increases the elastic modulus, several factors related to our specific experimental conditions may have influenced the observed reduction in Young’s modulus of glycated collagen. First, in our experiments, we used samples of early glycated collagen. This process likely induces specific structural alterations in the collagen molecule like Amadori products, preceding the accumulation of AGEs, which makes collagen more hydrophilic [17]. The increased hydrophilicity leads to weaker molecular interactions, thus reducing collagen stiffness and potentially explaining the observed reduction in Young’s modulus (reduced stiffness). Secondly, the experiments were performed using collagen solutions, which may behave differently from native collagen matrices utilized for AGE elasticity investigation [1,7]. Consistently with our observations Vaez et al. [1] also reported a decrease in the elastic modulus of individual collagen fibrils (within a scaffold) and single collagen fibrils due to glycation, despite an overall increase in the elastic modulus of collagen scaffolds. Additionally, incubation at 37 °C may induce changes in the collagen structure, impacting its mechanical properties, as various environmental factors can influence collagen integrity. This underscores how experimental conditions (solution vs. scaffold) significantly affect collagen’s mechanical response to glycation.

The study suggests a complex interplay between multiple adhesion mechanisms, potentially involving both integrin-dependent pathways and the receptor for advanced glycation end products (RAGE). The rapid initial adhesion to glycated collagen, particularly under flow conditions, suggests that integrin-dependent attachment can occur much faster than previously thought or that alternative mechanisms, such as RAGE-mediated adhesion, play a more significant role than currently understood. These findings have significant implications for tissue engineering and regenerative medicine, particularly in contexts involving diabetic or aging patients where glycated collagen is prevalent. The study also highlights the importance of considering dynamic conditions in cell–matrix interaction studies, as the dramatic differences between static and flow conditions suggest that traditional static assays may not fully capture the complexity of in vivo cell behavior.

## Figures and Tables

**Table 1:** Surface properties of collagen, summarizing roughness values (Rrms), surface potential, Young’s modulus (Ea), and adhesive forces determined for native collagen (Nat), collagen glycated for 1 day (GL1), and collagen glycated for 5 days (GL5).

Collagen Samples	Rrms (nm)	Surface Potential (mV)	Ea (MPa)	Adhesive Forces (nN)
Nat	3.01 ± 0.4	789 ± 93.7	34.8 ± 5.4	57.5 ± 0.6
GL1	2.53 ± 0.3	559 ± 18.4	6.90 ± 0.5	31.14 ± 0.3
GL5	7.70 ± 0.6	659 ± 8.8	2.07 ± 0.3	17.06 ± 0.2

## Data Availability

The original contributions presented in this study are included in the article. Further inquiries can be directed to the corresponding author.

## Abbreviations

Adipose-derived mesenchymal stem cells (ADMSCs), matrix metalloproteinases (MMPs), rat tail tendon collagen (RTC), 1-day glycated collagen sample (GL1), 5-day glycated collagen sample (GL5), fluorescein diacetate (FDA), root mean square roughness (Rrms), transforming growth factor-β (TGF-β), advanced glycation end products (AGEs), receptor for advanced glycation end products (RAGEs), melanoma cell adhesion molecule (MUC18), activated leukocyte cell adhesion molecule (ALCAM), trinitrobenzene sulfonic acid (TNBS). RGD and GFOGER, are sequences of amino acids, abbreviated as follows: D—aspartate, G—glycine, E—glutamate, R—arginine, O—hydroxyproline.
